# Report of the Committee to Urge the Enactment of Suitable Laws by the State Legislature

**Published:** 1867-07

**Authors:** 


*To the Honorable President and Members of the Illinois State Medical Society:—*

The committee, to whom was entrusted the draft for the afore-mentioned law, would beg leave to report:—

That prior to the session of the General Assembly, ending the current year, there was a correspondence opened with several legislators whose opinions were favorable to the scheme, and the draft submitted to this body one year ago, at Decatur, by the Macon County Medical Society, was sent to them. There was, therefore, a very favorable element in quite a number of intelligent men of both brancues of the Legislature anxious for the passage of such an act.

About ten days after the assembling of the Legislature, a quorum of this committee met in Springfield and consulted as to a plan of procedure, and determined that the bill should make its appearance in the Senate first. It was, therefore, presented to that body by its able and zealous friend, the Hon. W. H. Cheney, of McLean Co. We had a preference that it should be referred—because of its pertinence—to the Committee on Education, which preference met the approval of the honorable senator presenting it. We consulted the members of the Committee on Education, in part, concerning the prospects of the passage of the bill, and found no opposition to it after they saw fully what it assumed to correct.

Starting it thus in the Senate, and feeling favorably impressed with the encouraging assurances which we received from various influential senators, we next consulted with many members of the House of Representatives concerning its passage through that body, should it succeed in passing through the Senate, and for a time felt sanguine of success, from the promises we received. But the golden pitcher was broken at the *fountain.* The Committee on Education tabled the bill in their room, and thus the thing was *still-born*, or rather still *unborn*, and so remained. Our honorable senator attempted to take it from the table that it might be recommitted, but it failed even in that.

There was so determined a spirit in the House that something of the kind should pass, that a bill was prosented to that body, bearing features widely differing from those which this committee was ordered to present, and considered by its friends as more conservative, which, although not killed in the committee rooms, was in the House.

It is, however, flattering, to reflect that the necessity of such a law was entertained by the Senate and House, by a larger number of men than any similar bill had ever been before. We therefore hope and expect that the intelligence of our lawmakers will so grow that to pass a statute possessing the plain-faced merit of the one which we presented will, some day soon, be the accomplishment of our continued and persistent effort. We therefore recommend that the enterprise be continued, by the appointment of a more weighty and politically influential lobbying committee.

S. T. TROWBRIDGE, M.D.

GEO. T. ALLEN, M.D.

P. H. BAILHACHE, M.D.

Dr. W. S. Edgar offered the following, which was adopted:—

*Resolved*, That a committee of five be appointed, to report a memorial and law, to be presented to the Legislature at its next regular session, for the better regulation of the practice of medicine in the State; said committee to report the result of their labors at the next meeting of the Society.

